# The chemical brain hypothesis for the origin of nervous systems

**DOI:** 10.1098/rstb.2019.0761

**Published:** 2021-03-29

**Authors:** Gáspár Jékely

**Affiliations:** Living Systems Institute, University of Exeter, Stocker Road, Exeter EX4 4QD, UK

**Keywords:** nervous system evolution, neuropeptide, placozoa, sponge, ctenophore, cnidaria

## Abstract

In nervous systems, there are two main modes of transmission for the propagation of activity between cells. Synaptic transmission relies on close contact at chemical or electrical synapses while volume transmission is mediated by diffusible chemical signals and does not require direct contact. It is possible to wire complex neuronal networks by both chemical and synaptic transmission. Both types of networks are ubiquitous in nervous systems, leading to the question which of the two appeared first in evolution. This paper explores a scenario where chemically organized cellular networks appeared before synapses in evolution, a possibility supported by the presence of complex peptidergic signalling in all animals except sponges. Small peptides are ideally suited to link up cells into chemical networks. They have unlimited diversity, high diffusivity and high copy numbers derived from repetitive precursors. But chemical signalling is diffusion limited and becomes inefficient in larger bodies. To overcome this, peptidergic cells may have developed projections and formed synaptically connected networks tiling body surfaces and displaying synchronized activity with pulsatile peptide release. The advent of circulatory systems and neurohemal organs further reduced the constraint imposed on chemical signalling by diffusion. This could have contributed to the explosive radiation of peptidergic signalling systems in stem bilaterians. Neurosecretory centres in extant nervous systems are still predominantly chemically wired and coexist with the synaptic brain.

This article is part of the theme issue ‘Basal cognition: multicellularity, neurons and the cognitive lens’.

## 1. Introduction

Many theories have been put forward to explain nervous system evolution. The different theories often focus on the evolution of some salient aspects of the brain. These include the evolution of electrical conduction [1], the origin of spiking and voltage-gated channels [2,3], the diversification of neuronal cell types [4], the patterning of the nervous system along the main axes of the body [5,6], the development of neuronal elongations and synaptic circuits [7], the origin of sensory capacities [8,9] or the internal coordination of muscles [10]. These various theories are often complementary and attempt to give an account of nervous system origins from different angles [11].

Here, I approach the question by asking how the first proto-neurons organized into cellular networks with the rapid propagation of excitation. This excludes ionic flows in broad electric fields as occurs in bioelectric signalling during development and regeneration [12,13].

In neural networks, cellular excitation can propagate between cells by different mechanisms. During synaptic transmission, one cell influences the activity of others through chemical or electrical synapses. In ephaptic coupling, extracellular currents generated by one neuron directly alter the excitability of adjacent neurons (field effects) [14–16]. A third mechanism is volume transmission mediated by diffusible chemical signals linking signal-secreting sender cells to receptor-expressing receiver cells. Both chemical and synaptic transmission can wire complex neuronal networks with specific connections while specificity is more limited in ephaptic coupling.

Here I propose a detailed hypothesis, the chemical brain hypothesis for nervous system origins. The theory suggests that the first cellular networks involved in sensing, reacting and coordination of tissue-level and whole-body activity were organized by paracrine signalling. As signalling molecules, I will discuss the potential early origin and function of secreted neuropeptide-like molecules (in short, neuropeptides). I will also discuss several predictions of the hypothesis that can be tested experimentally.

The general idea that volume transmission may have evolved before synaptic transmission has been proposed by Grundfest, Horridge and others (reviewed in refs. [17,18]).

In §2, I give an overview of peptidergic signalling and the diversity of peptidergic systems in Metazoa. In §3, I define the chemical brain hypothesis and discuss the possibilities and constraints of peptidergic signalling to organize cellular networks. In §4, I discuss cellular transition scenarios for nervous system origins, in light of the hypothesis. Finally, I discuss some predictions of the hypothesis and how they could be tested.

## 2. The diversity and ancestry of peptidergic signalling in Metazoa

Neuropeptides are abundant, diverse secreted intercellular signalling molecules, which are near-ubiquitous in nervous systems. The active signalling peptides are produced in the Golgi from larger propeptides through successive steps of proteolytic cleavages and further chemical modifications. Cleavages generally occur at di- or monobasic cleavage sites (e.g. KR) by prohormone convertases. The cleaved peptides can be further modified, most often by C-terminal α-amidation during which the bifunctional peptidylglycine α-amidating monooxygenase (PAM) enzyme converts a C-terminal glycine into an amide [19]. Mature peptides travel in secretory vesicles called dense-core vesicles and are released at synapses or at non-specialized release sites along neurites. Release is regulated by intracellular cAMP and calcium levels [20–22]. The machineries for the acidification and release of dense-core vesicles and synaptic vesicles have many shared but also unique components [23–25]. Secreted peptides diffuse as paracrine signalling molecules or are transported by the bloodstream if the release occurs in neurohaemal organs (e.g. vertebrate pituitary). Neuropeptides act through cell surface receptors, most commonly G-protein coupled receptors (GPCRs).

### (a) Comparative genomics of peptidergic signalling systems

Comparative genomic studies of neuropeptides and their receptors have recently clarified the global patterns of the evolutionary diversification of peptidergic systems across animals [26–30]. Neuropeptides are present in all metazoans with the exception of sponges where none have yet been identified. Some of the neuropeptide families show deep conservation across the animal tree and trace back to the eumetazoan or cnidarian–bilaterian common ancestor. Ctenophores (sea gooseberries, representing the sister group to all other eumetazoans [31]) have many different neuropeptide molecules with no recognizable relationship to other metazoan peptides [29]. Only one of these is shared with other eumetazoans, the extracellular signalling molecule Trunk (a paralog of prothoracicotropic hormones) [32]. At least six peptide families trace back to the cnidarian–bilaterian common ancestor (RFamide, VWamide, PRXamide, insulin-like peptides, eclosion hormone (EH), bursicon) [32–35]. These in general show a many-to-many relationship to bilaterian peptide and receptor families. For example, a group of cnidarian receptors including the *Clytia hemisphaerica* maturation-inducing hormone receptor (MIHR) is sister to a bilaterian clade containing receptors for luqin, NPF, QRFP, tachykinin, FMRFa and NPY peptides [30,33]. Placozoans (millimetre-sized flat animals with no muscles or neurons) also contain several neuropeptides [26,32,36,37]. The bilaterian common ancestor had at least 30 neuropeptide-receptors systems and these show general conservation across major bilaterian clades with patterns of losses and further clade-specific divergences [26–28,30,38].

These phylogenetic patterns are in agreement with an ancestral core set of peptide–receptor pairs and the independent diversification of peptidergic signalling systems in cnidarians, bilaterians and ctenophores.

### (b) Peptidergic signalling can wire complex cellular networks

**Figure 1.**
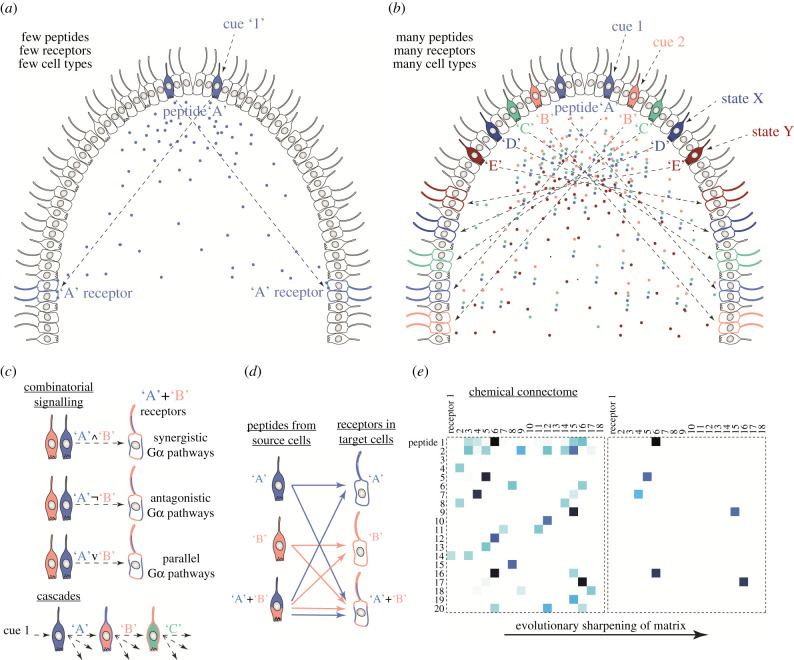
Wiring of complex networks in pre-nervous systems. (*a*) A hypothetical early animal with a ciliated epithelium with interspersed locomotor and sensory cells. One cell type expresses a signalling peptide that is released upon an external cue. The peptide signals to another cell type expressing a specific receptor for the peptide. (*b*) Through the diversification of cell types, signalling peptides and their receptors, an organism with more complex chemical wiring evolves. Note that peptides can be regulated both by external cues and internal states (e.g. autonomous activity, circadian rhythm, hunger, pacemakers). The effector systems could also include contractile cells. (*c*) If combinatorial signalling is possible, the chemical networks can encode state/cue combinations. This requires synergistic or antagonistic intracellular pathways (through Gα proteins for GPCRs). (*d*) With two peptides and two receptors it is possible to have eight signalling links with potentially different signalling outcomes. (*e*) The chemical connectome represents the matrix of ligand–receptor coupling but also the cellular coupling through these signalling pairs. Following gene duplications, the chemical matrix also evolves, e.g. through increasing specificity.

In nervous systems, there are two ways to build intercellular networks of signalling. In synaptic networks, cells or their processes connect to proximal cells via chemical or electric (gap junction) synapses. In peptidergic (or other paracrine) networks, ‘sending’ peptide-expressing cells connect to ‘receiving’ receptor-expressing cells [39]. In such chemical networks, links are defined by ligand–receptor specificity and by the pattern of ligand and receptor expression. If there are many signalling peptides and receptors, it is possible to wire complex cellular networks by peptidergic signalling alone. The co-expression of multiple propeptides or multiple receptors in the same cell allows the further, combinatorial diversification of signalling (figure 1). If two signalling peptides (*pep_1_* and *pep_2_*) are released by the same cell, these could act on three different types of target cells, one expressing a receptor for *pep_1_*, one for *pep_2_*, and one for both. With two peptides and two receptors, it is possible to wire a network with 8 possible connections, each linking a different subset of cells and with potentially different signalling consequences (figure 1*d*). If the second messenger cascades of the receptors are different and act synergistically or antagonistically, this could lead to multiple different signalling outcomes (figure 1*c*). The system can thus have multiple states of activity that could encode several external or internal states.

Complex paracrine networks wired by neuropeptide and monoamine signalling indeed exist and have been mapped in several bilaterian nervous systems, including *Caenorhabditis elegans*, a nematode [39], *Platynereis dumerilii*, an annelid [40], *Drosophila melanogaster*, an arthropod [41], and *Mus musculus*, a vertebrate [42].

The high diversity and cell-type-specific expression of neuropeptides in the non-bilaterian lineages of placozoans, cnidarians and ctenophores [29,37,43–45] also suggests the presence of specific cell-to-cell signalling and complex peptide-wired cellular networks in these organisms. In the placozoan *Trichoplax adhaerens*, for example, there is a highly cell-type-specific neuropeptide expression with over 10 neuropeptides each expressed in distinct cells, as shown by co-immunolabelling [36] and single-cell transcriptomics [46].

The evolution of cell-type-specific expression of peptides and receptors necessitates a unique and heritable gene regulatory landscape for each cell type. The diversification of chemical networks thus required not only the diversification of peptides and their receptors, but the diversification of cell types expressing unique combinations of them [47]. The two could have been strongly coupled in a sense that a new cell type (e.g. following sensory diversification) had to distinguish itself by a new chemical signature (i.e. a peptide mix). The tight specificity link between neuronal cell types and neuropeptides seems to be a general principle. In the mouse neocortex, neuropeptides and their receptors are ‘exceptionally potent neuron-type markers’ [42]. Similar observations were made in the larval *Platynereis* brain based on single-cell RNAseq data [40]. Neuropeptides seem to be among the most specific and most highly-expressed neuronal markers across animals. This suggests that each neuron type has a specific peptidergic fingerprint. Upon activation, this fingerprint reveals the identity of the cell to its neighbours by paracrine signalling. The chemical brain hypothesis states that this and not the language of synapses is the first language proto-neurons used.

## 3. The chemical brain hypothesis

This section starts by introducing the main postulates of the chemical brain hypothesis. I will then explore why peptides became the most abundant intercellular signalling molecules in animal nervous systems. I also discuss how peptidergic signalling can wire complex cellular networks.

### (a) Formulation of the hypothesis

The chemical brain hypothesis posits that elementary nervous systems first evolved as chemically connected networks of excitable cells (figure 1). This idea shows some parallels to the metabolism-first (as opposed to genetics-first) scenarios for the origin of life [48–50]. In chemical nervous systems, there were no synapses yet and cellular patterns (e.g. waves) of excitation propagated by the release of secreted signalling molecules that influenced the activity of target cells expressing specific receptors. Cellular excitation here refers to nonlinear changes in the cell's ionic or second messenger (e.g. cAMP) content playing out on the millisecond or second timescale. Such excitation can be elicited by both ionotropic and metabotropic receptors and can lead to cellular responses (e.g. contraction). The signalling molecules may have been small molecules (e.g. glutamate, GABA, NO, ATP) and small secreted peptides. Owing to their unlimited potential to diversify, peptides became the most significant paracrine signalling molecules. Peptides signalled environmental or internal states and enabled the coordination of effector activity and physiology in multicellular animal bodies. Paracrine signalling made chemical nervous systems diffusion limited, suggesting that they could only have worked efficiently in small organisms. To overcome the limitations of diffusion, peptidergic cells evolved cellular projections, the precursors to axons, to increase the available surface for secretion. Synapses may have first evolved to link cells expressing the same peptides into neuronal nets allowing coordinated release of peptides through synchronization. As animals grew bigger, synaptic signalling started to dominate and spread to the control of effectors. The evolution of circulatory systems in stem Bilateria enabled the rapid body-wide transport of peptides from neurohaemal release sites, overcoming the diffusion barrier. In parallel, peptidergic systems underwent explosive radiation. Complex peptidergic signalling networks still occur in every nervous system and modulate every circuit. These chemical networks form several hidden layers in the multilayer connectome of all animal nervous systems where synaptic connectivity represents only one of many layers.

### (b) Small peptides as the most successful neuronal signalling molecules

Signalling neuropeptide-like molecules feature prominently in the chemical brain hypothesis. Their diversity and phylogenetic ancestry makes them the most likely molecules to have wired chemical networks in early animals. Neuropeptides are highly diverse and are present in all major clades of animals, with the exception of sponges [26–28,36,37,51,52]. Other signalling molecules could also have had an early origin in animal cell–cell communication, including GABA [53], glutamate, monoamines and nitric oxide (NO), a gaseous paracrine signalling molecule. NO signalling is present in sponges [53], placozoans [54], cnidarians [55], ctenophores [56] and bilaterians [57]. However, since NO chemistry lacks variation, this system could not have diversified into multiple related signalling molecules. Monoamines (serotonin, dopamine, noradrenaline, octopamine etc.) are very important in bilaterian nervous systems, but likely only diversified in the bilaterian stem group [58]. Their pre-bilaterian origins and functions are unclear.

By analogy with ecology, one can evaluate the success of a class of molecules as one can evaluate the success of a phylogenetic clade: by species richness and per cent cover (e.g. [59]). According to these measures, neuropeptides are the most successful signalling molecules. They outnumber classical neurotransmitters by at least an order of magnitude in most nervous systems [28,30,43,60–64]. In terms of cover, neuropeptides collectively also rival classical neurotransmitters as they occur in most if not all neurons, often co-occurring with small transmitters [65,66]. Even in the mammalian neocortex—the epitome of a synaptically connected structure—almost all neurons express one or more neuropeptides and neuropeptide receptors [42]. Several neuropeptides are also widely expressed in the central nervous system of cephalopods [67–70].

Why did peptide signalling molecules attain such high diversity in nervous systems? Why were peptides favoured in evolution over small molecules (e.g. NO, GABA) or globular proteins to wire chemical cellular networks? To address this question, we can compare these different classes of molecules in terms of their cost to the cell, their potential for evolutionary diversification, their diffusibility, stability and other measures (table 1).

**Table 1.** Characteristics of various classes of neuronal signalling molecules. See main text for references.

type of signalling molecule	diffusion coefficient	synthesis	evolvability	diversity
ions, gases (NO)	∼1–2 × 10^−5^ cm^2^ s^−1^	n.a. or by NO synthase	none	limited
small molecules	∼2 × 10^−5^ cm^2^ s^−1^	synthesis requires several specific enzymes	limited, requires the evolution of new enzymatic activities, can co-evolve with receptor	theoretically unlimited, limited by evolutionary constraints
neuropeptides	∼2–5 × 10^−6^ cm^2^ s^−1^	on ribosomes, followed by proteolytic cleavage and modification	highly evolvable, by divergence within a multi-copy precursor, by gene duplication, often co-evolves with receptor	unlimited, 20*^n^*^,^ where *n* is sequence length (limited by solubility, stability)
globular proteins	∼2–10 × 10^−7^ cm^2^ s^−1^	on ribosomes	highly evolvable, by gene duplication and divergence	unlimited, 20*^n^*^,^ where *n* is sequence length (limited by folding, stability)

**Figure 2.**
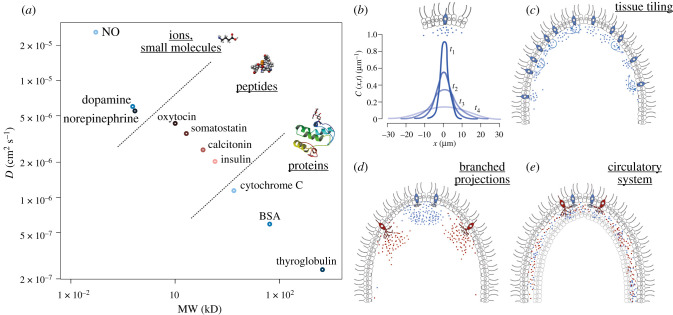
Diffu sion of macromolecules and the diffusion limitation in chemically wired nervous systems. (*a*) Relationship of molecular weight and diffusion coefficient. Examples of proteins, neuropeptides, catecholamines and a small molecule (nitric oxide, NO), compared for their molecular weight (MW) in kilodaltons and diffusion coefficient (*D*), log–log scale. BSA, bovine serum albumin. See main text for references. (*b*) Temporal change (from *t*_1_ to *t*_4_) of the concentration gradient by diffusion of signalling molecules after a local release event. The curves are only illustrations, based on the theory of diffusion, after [76]. (*c*) Tiling an epithelium with cells of the same type releasing the same peptide under the same conditions allows tissue-wide signalling. Autocrine signalling can allow spread and amplification. (*d*) Branched projections filled with dense-core vesicles increase the membrane area available for secretion allowing the release of more peptides per event. (*e*) Fluid-filled spaces can speed the spread of signalling molecules, especially when aided by active circulation.

In terms of costs to the cell, short peptides are cheaper than long globular proteins. Several similar small peptides (even over 30) can also be produced by the cleavage of one precursor. In terms of diffusivity, small peptides and small molecules are generally more diffusive than globular proteins. The diffusion coefficient *D* of a molecule is proportional to its molecular mass as *D* ∼ *M*^−0.3^ [71,72]. For example, the *D* of bovine serum albumin (*M* = 65 kD) is approximately 5 × 10^−7^ cm^2^ s^−1^. For the nonapeptide oxytocin (*M* = 1 kD), the *D* is approximately 4.3 × 10^−6^ cm^2^ s^−1^ [73]. Catecholamines have a diffusion coefficient of approximately 5.5 × 10^−6^ cm^2^ s^−1^ (M) in water [74]. For nitric oxide (*M* = 30 dalton), *D* is 2.60 × 10^−5^ cm^2^ s^−1^ [75]. Figure 2 shows the relationship between molecular weight and diffusivity. Peptides clearly outperform proteins in their diffusibility, providing an advantage of faster spreading in paracrine signalling.

Next, we can compare the diversity of potential types evolution has access to within a class of molecules. Nitric oxide is monotypic and an evolutionary dead end. The diversity of small signalling molecules is theoretically endless, but their gradual diversification from one precursor (e.g. the origin of tyramine, octopamine, dopamine and noradrenaline from tyrosine) is limited. Furthermore, the evolution of a new version of a small molecule requires the evolution of a new enzymatic activity. This further limits the evolvability of small-molecule signalling pathways. In contrast, peptides have unlimited diversity, with a 5 amino-acid-long form having 20^5^ possible variants, not considering modifications (although solubility and stability will somewhat limit the number of variants). Peptides can also easily diversify through the process of gene duplication and divergence or by intra-precursor divergence [77]. The evolution of receptors can follow, through coevolutionary diversification (duplication of both ligand and receptor, followed by the divergence of specificity), a general process in the evolution of peptide–receptor systems [26–28].

Overall, if one considers synthesis costs, copy number, diffusibility, evolvability and potential diversity, small peptides are the clear winners and evolution did not overlook them.

A limitation of peptidergic systems is the lack of dedicated reuptake pathways. For classical neurotransmitters, specific reuptake pathways (e.g. SERT serotonin transporter) regulate the timing and extent of signalling. One possible mechanism to tune neuropeptide signalling is through the activity of extracellular proteases. In the vertebrate brain, membrane-bound or soluble proteases can cleave secreted neuropeptides to alter their activity or degrade them [78]. Peptidergic signalling is also slower than synaptic signalling and plays out in the second rather than millisecond timescale. The main, early limitation, however, was probably diffusivity.

### (c) Peptidergic nervous systems are diffusion limited

Peptidergic nervous systems are limited by diffusion. If, following activation, a cell releases a unit amount of signalling peptide, the peptide will diffuse with the characteristic diffusion constant in the intercellular space and its concentration will decay exponentially from the source and this curve will flatten with time (figure 2*b*). Depending on the initial concentration and peptide–receptor affinity (EC_50_ is often in the low nanomolar range for neuropeptide-GPCR activation), a peptidergic cell will only be able to signal to other cells within a given distance and its ability to reach more distant cells will decay exponentially.

There are at least three ways to overcome this diffusion barrier and to deliver signals to every cell in a tissue or across the entire organism. The first solution is to tile a surface (e.g. an epithelium) with several peptidergic cells of the same type (figure 2*c*). We can see this for example in placozoans, the ectoderm of cnidarians, or the gut of mice or *Drosophila* [79–81]. The placozoan *Trichoplax adhaerens* is tiled with a mosaic of peptidergic cells, most abundant at the perimeter of the disc-shaped body. The ectoderm of the anthozoan *Nematostella vectensis* contains nerve nets of tri- and quadripolar neurons expressing GLWamide [82]. GLWamide-expressing cells form similar nerve nets in hydrozoan polyps [83,84]. In the *Clytia* gonad, cells expressing the neuropeptide maturation inducing hormone (MIH) tile the entire epithelium. The gut of mice and *Drosophila* is tiled with diverse peptidergic enteroendocrine cells expressing various combinations of neuropeptides [80,81]. From these examples, we can also estimate a peptidergic cell's signalling range as a few cell diameters or a few 10s of µms (assuming uniform receptor expression). Autocrine signalling (the peptide stimulates its own release) can also lead to signal amplification and sustained and travelling activation. The second solution is to increase the concentration of secreted molecules by increasing the number of vesicles and the available membrane surface for secretion. This can be achieved by the development of branched projections (figure 2*d*). The third solution is to evolve a mechanism to deliver signalling molecules more rapidly across the body by active fluid circulation (figure 2*e*). I will discuss the evolutionary implications of the diffusion limit and the solutions to overcoming it in §4.

## 4. Peptidergic signalling and scenarios for nervous system origins

In this section, I discuss cellular transition scenarios for the origins of nervous systems, in light of the principles of paracrine signalling outlined above. I first review the evidence for the possible ciliary origins of neuropeptide signalling. I then discuss cases of neurosecretory signalling in cnidarians, placozoans and sponges. This is followed by a proposal for an elementary combinatorics of infraneuronal systems that helps to focus the discussion about when and in which order neuronal subsystems could have appeared and evolved into nervous systems. Finally, I turn to the origin of neuronal processes, synaptically connected networks and neurohaemal organs.

### (a) Ciliary origins of neuropeptide signalling

How did neuropeptidergic signalling originate in animal evolution? What were its precursors and potential initial functions? Recent work from the laboratory of Betty Eipper has provided fascinating insights into this question. In 2016, the Eipper lab reported the presence of the peptidylglycine α-amidating monooxygenase (PAM) enzyme in the green alga *Chlamydomonas reinhardtii* [85]. PAM is involved in the α-amidation of neuropeptides in animal nervous systems and its presence in a green alga was surprising. An earlier bioinformatic study showed that PAM occurs in all animals and also outside animals in some protist lineages, and its origin thus predates nervous systems [86].

The Eipper team found that PAM localizes to the cilia of *Chlamydomonas*. In subsequent work, they showed that PAM is required for the formation of cilia in *Chlamydomonas* [87]. In animals, the prime substrate of PAM are the cleaved, maturing neuropeptides. What could the enzyme modify in the alga? A mass-spectrometry screen revealed the identity of one of PAM's substrates in *Chlamydomonas* as a chemoattractant peptide released on ciliary ectosomes that attracts gametes of the minus mating type [88]. This beautiful work shows the unexpected deep evolutionary ancestry of the machinery to produce amidated peptides and shows that the products of this machinery in a green alga are involved in cell to cell signalling.

Comparative studies also suggest a widespread and ancient evolutionary connection of GPCR signalling to cilia. A proteomic analysis of cilia in the sea anemone *N. vectensis* and the sea urchin *Strongylocentrotus purpuratus* identified several GPCRs and GPCR signalling components localized to the cilium [89]. Several neuropeptide GPCRs also localize to primary cilia in mammals [90].

These findings suggest a scenario whereby a signalling machinery involved in ciliary communication in protistan ancestors was recruited during animal evolution for the processing of signalling neuropeptides involved in intercellular communication. The secretion of bioactive amidated peptides in ciliary ectosomes emerges as the most likely cellular mechanism from which metazoan neuropeptide signalling evolved. Early on, secretion may have been apical and the amidated peptide products of PAM may have acted on GPCRs expressed on the cilia of receiving cells. The secretory machinery was later redirected to the nascent processes of the proto-neurosecretory cells. The further elucidation of the origin of neuropeptide signalling will require the identification of the substrates of PAM in choanoflagellates and sponges.

### (b) Sensory-neurosecretory cell types at the origin of nervous systems

In what cellular and tissue context could have early peptidergic signalling operated? The studies of peptidergic systems in non-bilaterians can inform our thinking about early peptidergic networks.

Peptidergic signalling is best understood in the cnidarians among the non-bilaterians [91]. In many cnidarians, RFamide neuropeptides are localized to dense-core vesicles, as evidenced by immunogold electron microscopy [92–95]. Such peptidergic vesicles have been observed in planula larvae, polyps and medusae in diverse species and can occur at nonsynaptic or synaptic release sites, including neuromuscular synapses. Synapses containing clear synaptic vesicles are also present in the cnidarian nervous system (e.g. [96]) but no small molecule transmitter has yet been directly localized to such synapses.

Exogenous RFamide peptides can cause muscle contractions and seem to have a direct excitatory effect on muscles [97–99]. GLWamide and other neuropeptides can also induce muscle contractions in various cnidarians [83,91,100–102]. The RFamide peptides can signal through a family of trimeric peptide-gated ion channels (*Hydra* Na^+^ channel; HyNaCs) [103,104]. The presence of these ionotropic peptide receptors indicates that neuropeptides may act as fast neurotransmitters at least in some contexts in cnidarian nervous systems.

A particularly well-studied example of peptidergic signalling in a cnidarian is presented by light-sensory peptidergic cells in the hydrozoan *Clytia hemisphaerica*. These sensory-neurosecretory cells regulate the light-induced spawning of the *Clytia* jellyfish. The cells tile the gonad epithelium and express an opsin (a G-protein coupled receptor that together with retinal forms a photopigment) and a neuropeptide involved in oocyte maturation (maturation inducing hormone, MIH) [105]. An increase in ambient light (at dawn in a natural setting) leads to the release of MIH, a process that is defective in opsin mutant jellyfish [79]. The peptide activates a GPCR receptor (the MIH receptor) expressed in the oocytes to trigger spawning [33].

Neuropeptide-secreting cells with putative sensory functions have also been described in placozoans. Here, several distinct neuropeptide-like molecules, expressed in distinct cell types, can induce dramatic behavioural changes when added to the animals [36,106]. The behaviours include crinkling, rotation or flattening. Some of the peptide-expressing cells have a sensory morphology with a cilium [107]. It is not known which sensory cues may trigger peptide release, but some cues such as UV light trigger behaviours similar to the behaviours induced by some peptides [108]. This suggests that sensory cues could trigger the release of specific peptides from distinct cell types, eliciting coordinated responses. Peptides may also coordinate movements during autonomous behaviours (e.g. waves of epithelial contractions) [109].

Some sponges may also have sensory-neuroendocrine cells linking environmental cues to behaviour or developmental processes. Larvae of the demosponge *Amphimedon queenslandica* have different populations of flasked-shaped sensory cells that respond to settlement cues by calcium signalling [110], and release nitric oxide to regulate larval metamorphosis [111]. Although no neuropeptide-like molecule has been found in sponges, NO also represents a paracrine, diffusible molecule and the cells can be considered sensory-neuroendocrine.

### (c) Elementary combinatorics of infraneuronal systems

**Figure 3.**
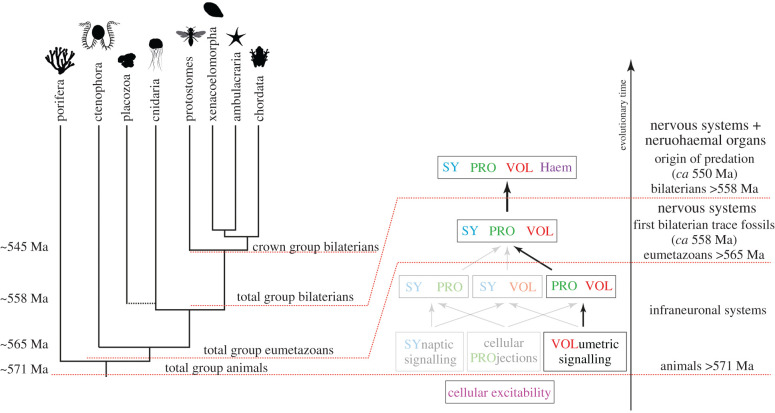
Elementary combinatorics of infraneuronal systems and the path suggested by the chemical brain hypothesis. Phylogenetic tree of major groups of animals under sponges-first, representing our best current understanding of animal phylogeny [31]. Total group animals appeared shortly before 571 Ma, as suggested by the fossil record [115], and inherited the property of cellular excitability from their protist ancestors. Some aspects of advanced nervous systems appeared before eumetazoans, including volumetric signalling and possibly cellular projections involved in signalling. Total group eumetazoans appeared somewhat before 565 Ma with nervous systems combining synaptic transmission, projections and volume transmission appearing in the stem lineage or independently in ctenophores and cnidarians + bilaterians. The first bilaterian trace fossils date to around the same time [116]. With total group bilaterians, neurohaemal organs and centralized brains started to evolve [117] around 558 Ma. This period experienced the great neuropeptide explosion and was followed by the origin of predation (*ca* 550 Ma) [3] and the Cambrian explosion. Images are from PhyloPic.

How did paracrine cellular networks evolve into synaptically connected nerve nets? When and why did projections and synapses appear and in what order? To explore this, I define an elementary combinatorics of infraneuronal systems for the origin of nervous systems, analogous to Szathmáry and colleagues' elementary combinatorics of infrabiological systems for the origin of life [112]. An infraneuronal system is defined as a necessary but not sufficient character of a structure that we would without doubt consider a nervous system. These infraneuronal systems include (i) cellular excitability, (ii) synaptic cell-to-cell signalling, (iii) cellular projections, and (iv) volumetric cell-to-cell signalling. Out of these four systems, cellular excitability through voltage-gated ion channels, pumps and receptors is the oldest and evolved in single celled organisms [113,114]. The various combinations of the three other characters define three possible pathways to a full-fledged nervous system (figure 3). As intermediates, we could imagine a nervous system with synapses + projections, volume transmission + projections, or synapses + volume transmission but no projections.

The chemical brain hypothesis proposes the early origin of neurosecretion, followed by the later evolution of projections and synapses. Below, I examine what could have favoured the origin of projections and synapses in an organism that already possessed neurosecretory cells.

### (d) Origin of neuronal projections

Why did neuronal projections first evolve? In a synaptically connected nervous system, the evolution of neuronal projections would allow long-range communication. Projections could either connect sensors to effectors [8,9] or contribute to the large-scale coordination of excitable tissue [7]. A computational model by de Wiljes and colleagues found that adding short projections that provide random connectivity can increase the coordination of activity patterns in larger tissues [7].

The chemical brain hypothesis offers an alternative explanation. In a peptide-secreting cell, branched cellular elongations containing secretory vesicles could increase the total membrane surface available for secretion. A cell with more projections filled with dense-core vesicles could secrete larger doses of peptides per excitation event, widening its range of signalling (figure 2*d*).

**Figure 4.**
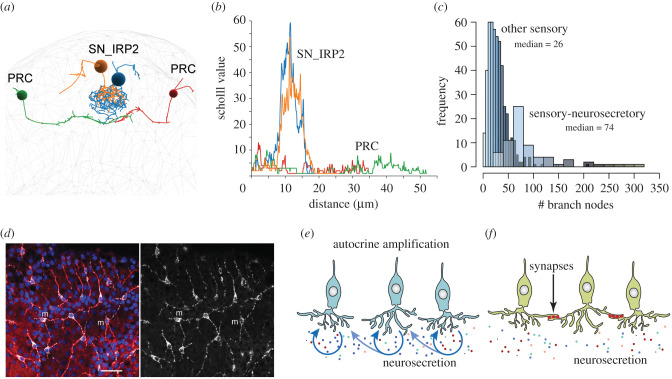
Neurite elongations in various neurosecretory cells. (*a*) Morphology of two synaptic photoreceptor cells (PRC) and two sensory-neurosecretory cells (SN_IRP2) secreting an insulin-related peptide and other peptides in the *P. dumerilii* larval brain. (*b*) Sholl analysis of the PRC and SN_IRP2 cells. A Sholl analysis scores the number of neuronal branches crossed by concentric circles of increasing radii centred in the soma of a neuron. (*c*) Histogram of the number of branch nodes in the *P. dumerilii* larval brain. The distribution is shown for sensory-neurosecretory cells and non-neurosecretory sensory cells. (*d*) Morphology of *Clytia* PRPamide-secreting cells. Confocal plane at the basal ectoderm level of the gonad. The anti-PRPamide staining labels peptidergic vesicles in the soma and processes of PRPamide cells (m). The anti-α-tubulin staining (red) highlights the microtubule bundles in the basal processes. Scale bar 20 µm. (*e*) Schematic of autocrine amplification of neurosecretion. (*f*) Schematic of synaptic amplification of neurosecretion. References: [40,79,118].

Neurosecretory cells can indeed have highly branched axonal morphologies and the branching neurites can contain many dense-core vesicles over their entire length. Figure 4 compares the morphology of sensory-neurosecretory neurons and non-neurosecretory sensory neurons in the anterior nervous system of larval *P. dumerilii* [40]. Comparing the number of branch points or individual neurons' Scholl value (a Scholl analysis scores how many times a neuron's branches cross concentric circles of increasing radii centred on the soma) shows that sensory-neurosecretory neurons are highly branched, more than other types of sensory neurons. Since many of the sensory-neurosecretory neurons lack classical synapses in *Platynereis* [40], their highly branched morphology likely evolved to maximize release surface.

Cellular projections could evolve to uniformly cover an area in the tissue, to ensure that similar levels of signalling peptides reach all target cells simultaneously. In the gonad ectoderm of the jellyfish *Clytia*, the MIH-expressing light-sensory cells have branched elongations that uniformly cover the tissue (figure 4*d*). The elongations are filled with neuropeptide-containing vesicles [79] (figure 4*d*). The tiling of the tissue together with the projections likely ensure a uniform release of MIH following a dark–light transition to uniformly stimulate the target cells, the maturing oocytes expressing the MIH receptor [33].

### (e) Origin of synapses

The origin of chemical synapses in nervous systems has been discussed by several authors. For example, Mackie discussed synapse origins in the context of myoepithelial sheets in which transmission first occurred through low-resistance cytoplasmic bridges. Later, these were replaced by synapses that provided an increased specificity of conduction [1].

The chemical brain hypothesis suggests an alternative path for the origin of synaptic connections. It may be that the first synapses evolved to connect several sensory-neurosecretory cells of the same type into neuronal nets. Synapses with activatory transmitters linking cells of the same type could have enabled synchronous activation, with coordinated pulses or travelling waves of activity. This could have ensured synchronized peptide release across the entire field of cells, contributing—together with the advantages provided by branched projections—to a more robust effector response.

This scenario predicts that a similar functional organization may still characterize some nerve nets in cnidarians. In such nerve nets, peptidergic cells could be linked by chemical synapses, employing small molecule transmitters, and effectors could be regulated by the synchronized paracrine release of neuropeptides. For example, in the *Clytia* gonad, the MIH-expressing cells may be linked through synapses. This could be tested by serial electron microscopy or by transgenic synapse markers. We know little about the nature of neurotransmitters in ctenophores and cnidarians and testing this scenario will require more research in this area. In *Hydra*, there are non-overlapping neuronal nets with distinct activity profiles [119]. What are the transmitters synchronizing the propagating waves of activity in these nets? What are the transmitters released to the effectors? RFamide peptides in *Hydra* can induce muscle contractions, acting through a large variety of peptide-gated channels (the *Hydra* Na^+^ channels or HyNaCs) expressed in epitheliomuscular cells and potentially involved in neuromuscular transmission [103].

From their function in synchronizing peptidergic networks, synapses may have spread into other cellular contexts. Owing to their more targeted, millisecond scale signalling eliciting spiking responses they started to dominate nervous system dynamics. This could have happened between 560–550 Ma as trace fossils started to diversify and animal-on-animal predation first appeared [3]. This also coincided with more complex, larger, and folded bodies (triploblasts) that presented a challenge for diffusion-limited paracrine signalling. One may think that this led to the demise of peptidergic networks. But this did not happen.

### (f) Origin of neurohaemal organs and the end-Ediacaran neuropeptide explosion

Comparative genomics indicates that peptidergic signalling systems have undergone an explosive radiation in stem bilaterians. There are approximately 30 proneuropeptide families and their receptors conserved across major bilaterian clades and most of these originated in the bilaterian stem [26–28,30,38]. Why did peptidergic systems diversify in stem bilaterians that already had the ability for fast synaptic signalling? If we look at the distribution of neuropeptides in bilaterian brains, we always find the highest diversity and concentration in anterior neurosecretory-neurohaemal organs where brain peptides are directly released into the haemolymph (e.g. centipedes [120], annelids [40], vertebrates [121]).

Neurohaemal organs where neurosecretory endings are in close contact with blood vessels have been described in many animals [117,122–124] including annelids (infracerebral complex) [125,126], molluscs [127] (e.g. the neurosecretory system of the vena cava in *Octopus*) [128], insects (pars intercerebralis–corpus cardiacum–corpus allatum system), crustaceans (X-organ and other organs) [124,129], millipedes [130,131], nemerteans [132], tunicates [123], cephalochordates [133] and vertebrates (the various circumventricular organs) [123].

The final postulate of the chemical brain hypothesis is that the evolution of circulation and neurohaemal organs released the constraints imposed on peptidergic signalling by diffusion. Hemocoelar circulation coupled to the release of peptides at a neurohaemal site ensured the rapid spread of peptides across the body.

Could it be that circulatory systems actually evolved for the transport of neuropeptides and not for the transport and exchange of gases and nutrients? Animals smaller than August Krogh's critical dimension of approximately 1 mm can rely on diffusion and skin breathing alone for respiration. Bilaterians in this size range can already have a haemocoel and active circulation, as found for example in the small interstitial annelid *Dimorphilus* (previously *Dinophilus*) *gyrociliatus* [134]. If gas exchange is not diffusion limited in an organism of this size, why does it have circulation? Could the reason be to ensure that signalling peptides reach target cells across the body to coordinate whole-body actions and physiology? There are many peptides expressed in the nervous system of *D. gyrociliatus* [135]. A neurohaemal organ has not been described but such organs have been studied in other annelids [125,126,136].

Neurohaemal organs thus allowed concentrated peptide release, setting free the peptides to travel in the ‘Loop’ train of the circulating haemolymph, defying diffusion. This new route to spread may have facilitated diversification, allowing peptides to remain on centre stage in bilaterian brains.

## 5. Testable predictions of the hypothesis

The chemical brain hypothesis discusses how elementary nervous systems may have functioned and evolved. Some of the ideas may apply to extant nervous systems, in particular to non-bilaterians and larval bilaterians that potentially retained a richer mosaic of ancestral characters. Evolutionary hypotheses and transition analyses try to account for past events but also aid thinking and hopefully stimulate future work. Future results based on predictions of the hypothesis can in turn test the hypothesis. Below I list some questions that were inspired by writing this piece and that could be tested experimentally.

What is the function of α-amidation in choanoflagellates and sponges? Are there amidated products involved in intercellular signalling? Are these in the gametes or multicellular stages? A better understanding of PAM function and related molecules (e.g. its copper transporter) could illuminate this.

Why do placozoan peptidergic cells have no projections? Fibre cells do have projections, thus the organism has the ability to grow them [107]. Could the reason be that at the scale of *Trichoplax*, diffusion is not limiting? Is the fluid-filled lumen between the dorsal and ventral epithelia a mediator of peptide signalling? Some of this could be tested using fluorescent tracers (e.g. dextrans, peptides) and live imaging.

Is there combinatorial and autocrine peptide signalling in placozoans and cnidarians? Are there cells coexpressing more than one peptide receptor? Could some of the ciliary-localized GPCRs in *Nematostella* function as peptide receptors? How complex are the peptidergic networks and do they contain peptide cascades? Addressing these questions will first require the identification and cellular mapping of such receptors.

What is the relationship between peptidergic and classical transmitter action in cnidarians? Are peptides or classical transmitters the main transmitters on effector cells? Which transmitters synchronize each of the non-overlapping neuronal networks in cnidarians [119]? Are there synapses between the MIH-expressing cells in *Clytia*? We will need to learn more about synapses and transmitters in cnidarians. New transgenic approaches [137,138] and serial EM could help to address this.

What is the primary function of circulation in small aquatic bilaterians? Is it the transport of oxygen, nutrients or hormones? Can we test this by optogenetically inhibiting the heart in a small interstitial animal? Would they suffocate or break down hormonally first?

More experimental work on ctenophores (sea gooseberries)—the sister group to all other eumetazoans [31]—would also be immensely useful for understanding nervous system origin(s) [18,29,51,56,107]. Ctenophores notoriously lack most classical neurotransmitters (except glutamate and GABA, but GABA is in muscles [139]) but express a large diversity of enigmatic neuropeptide-like molecules [29]. What are the functions of these peptides? What are their receptors? Are there peptide-gated channels in ctenophores, like in *Hydra* and some bilaterians [77,103,140]? Is the ctenophore nervous system wired into complex peptidergic cellular networks? How do peptidergic systems work together with glutamatergic signalling?

## 6. Envoi

Is the chemical brain hypothesis a good hypothesis about the origin of nervous systems? A hypothesis is good and useful if it has testable predictions and stimulates fresh thinking, new questions or experiments. As Raymond Goldstein put it, ‘If [physical] theories are crafted the right way they have utility even if proven wrong, sometimes especially if proven wrong!’ [76]. The same is true for macroevolutionary hypotheses. Writing this piece suggested new questions and experiments and hopefully will also stimulate the reader.

## Data accessibility

This article has no additional data.
